# The First Pilot Epigenetic Type Improvement of Neuropsychiatric Symptoms in a Polymorphic Dopamine D2 (-DRD2/ANKK (Taq1A)), OPRM1 (A/G), DRD3 (C/T), and MAOA (4R) Compromised Preadolescence Male with Putative PANDAS/CANS: Positive Clinical Outcome with Precision-Guided DNA Testing and Pro-Dopamine Regulation (KB220) and Antibacterial Therapies

**DOI:** 10.4236/oji.2024.143006

**Published:** 2024-09

**Authors:** Kenneth Blum, Igor Elman, David Han, Colin Hanna, David Baron, Ashim Gupta, Shan Kazmi, Jag Khalsa, Debasis Bagchi, Thomas McLaughlin, Rajendra D. Badgaiyan, Edward J. Modestino, Drew Edwards, Catherine A. Dennen, Eric R. Braverman, Abdalla Bowirrat, Keerthy Sunder, Kevin Murphy, Nicole Jafari, Foojan Zeine, Paul R. Carney, Mark S. Gold, Kai-Uwe Lewandowski, Alireza Sharafshah, Aryeh R. Pollack, Panayotis K. Thanos

**Affiliations:** 1 Division of Addiction Research & Education, Center for Sports and Mental Health, Western University of Health Sciences, Pomona, CA, USA; 2 Department of Psychiatry, Boonshoft School of Medicine, Wright State University and Dayton VA Medical Centre, Dayton, OH, USA; 3 Division of Nutrigenomics, Victory Nutrition International, Lederoch, PA, USA; 4 Institute of Psychology, ELTE Eötvös Loránd University, Budapest, Hungary; 5 Department of Psychiatry, School of Medicine, University of Vermont, Burlington, VT, USA; 6 The Kenneth Blum Behavioral & Neurogenetic Institute, LLC., Austin, TX, USA; 7 Department of Molecular Biology, Adelson School of Medicine, Ariel University, Ariel, Israel; 8 Division of Neuromodulation Research, Karma Doctors & Karma TMS, Palm Springs, CA, USA; 9 Division of Personalized Interventions, Peak Logic, Del Mar, CA, USA; 10 Division of Personalized Medicine, Cross-Cultural Research & Educational Institute, San Clemente, CA, USA; 11 Awareness Integration Institute, San Clemente, CA, USA; 12 Division of Personalized Pain Therapy, Center for Advanced Spine Care of Southern Arizona, Tucson, AZ, USA; 13 Department of Psychiatry, Harvard School of Medicine, Cambridge, MA, USA; 14 Department of Management Science and Statistics, University of Texas, San Antonio, TX, USA; 15 Behavioral Neuropharmacology and Neuroimaging Laboratory, Clinical Research Institute on Addictions, Department of Pharmacology and Toxicology, Jacobs School of Medicine and Biomedical Sciences, University at Buffalo, Buffalo, NY, USA; 16 Future Biologics, Lawrenceville, GA, USA; 17 Department of Microbiology, Immunology, & Tropic Diseases, School of Medicine, Georgetown University, Washington DC, USA; 18 Department of Pharmaceutical Sciences, College of Pharmacy, Texas Southern University, Houston, TX, USA; 19 Department of Psychiatry, School of Medicine, Case Western University, Cleveland, OH, USA; 20 Brain and Behavior Laboratory, Curry College, Milton, MA, USA; 21 The Neurogenesis Project, Jacksonville, FL, USA; 22 Department of Family Medicine, Jefferson Health Northeast, Philadelphia, PA, USA; 23 Department of Psychiatry, UC Riverside School of Medicine, University California, Riverside, CA, USA; 24 Department of Human Development, California State University at Long Beach, Long Beach, CA, USA; 25 Department of Health Science, California State University at Long Beach, Long Beach, CA, USA; 26 Division Pediatric Neurology, School of Medicine, University of Missouri, Columbia, MO, USA; 27 Department of Psychiatry, School of Medicine, Washington University, St. Louis, MO, USA; 28 Cellular and Molecular Research Center, School of Medicine, Guilan University of Medical Sciences, Rasht, Iran

**Keywords:** PANDAS, CANS, Genetic Addiction Risk Testing (GARS), Pro-Dopamine Regulation, Hypodopaminergia, Polymorphisms, Antibacterial Therapy, Infections

## Abstract

Pediatric autoimmune neuropsychiatric disorders associated with or without streptococcal and other bacterial infections (PANDAS/CANS) are emerging as a featured pediatric disorder. Although there is some controversy regarding treatment approaches, especially related to the behavioral sequelae, we have hypothesized in other published work that it is characterized by the rapid onset of Reward Deficiency Syndrome (RDS) in children. We propose utilizing a multi-systems biological approach involving the coupling of genetic addiction risk testing and pro-dopamine regulation (KB220/POLYGEN^®^) to help induce “dopamine homeostasis” in patients with PANDAS, especially those with known DNA-induced hypodopaminergia. This case study examines a 12-year-old Caucasian male with no prior psychiatric issues who presented with a sudden onset of severe anxiety, depression, emotional liability, and suicidal ideation. The patient underwent genotyping and the genetic addiction risk score (GARS) testing, which revealed risk polymorphisms in the dopamine D2 (-DRD2/ANKK (Taq1A), OPRM1 (A/G), DRD3 (C/T), and MAOA (4R) genes. These polymorphisms have been linked to hypodopaminergia. The patient was subsequently placed on research ID-KB220ZPBMPOLY (POLYGEN^®^), and albeit the possibility of bias, based upon self and parental assessment, a marked rapid improvement in psychiatric symptoms was observed. In the second phase of treatment (102 days utilizing KB220), the patient received standard antibody testing, which was positive for Lyme. Antibacterial therapy started immediately, and KB220z was discontinued to provide a wash-out period. A monotonic trend analysis was performed on each outcome measure, and a consistently decreasing trend was observed utilizing antibacterial therapy. Our recommendation, albeit only one case, is to utilize and further research a combined therapeutic approach, involving precision-guided DNA testing and pro-dopamine regulation along with antibacterial therapy, as well as glutathione to address offensive enhanced cytokines, in patients with suspected PANDAS/CANS.

## 1. Introduction

Pediatric autoimmune neuropsychiatric disorders associated with streptococcal infections, Lyme, and other bacterial infections (PANDAS) are emerging as a featured pediatric disorder whereby a subset of children, especially in preadolescence, have a rapid onset of Reward Deficiency Syndrome (RDS) [1] [2], which includes obsessive-compulsive disorder (OCD), anxiety, depression, avoidant behaviors, and/or tic disorders. Additionally, learning regression, deterioration in handwriting, night-time fears, emotional lability, and separation anxiety have been documented in affected children. Dopamine function appears to play a significant role in RDS. However, dopamine is just one of several critical neurotransmitters in the mesolimbic/prefrontal cortex and brainstem, each playing an important role in managing behavior [3]. In unpublished work from Dr. Chris Turnpaugh’s, Health & Wellness Center (Mechanicsburg, PA) and others, certain foods like mint could affect both d1 and d2 receptor integrity.

These RDS and other mood behaviors may arise from group A beta-hemolytic streptococcal (GABHS) infections [1] [2]. Lyme and Borrelia, as well as other GABHS infections, can induce an autoimmune reaction and concomitant antibodies that interfere with normal brain function, specifically targeting mesolimbic dopaminergic loci [2]. Previously, our group suggested that Lyme disease’s principal vector in the United States is Ixodes scapularis (deer or black-legged ticks) and that those infected may present depression and anxiety, possibly due to hypodopaminergia [4]. Transcripts coding for two putative cytosolic sulfotransferases were also identified in this study, which demonstrated that these ticks recognized phenolic monoamines as their substrates. Specifically, sulfotransferase activity was found in later recombinant proteins against the neurotransmitters dopamine and octopamine. Furthermore, it was demonstrated in the salivary glands of Ixodid ticks that the activation of Ixosc Sult 1 and Sult 2 may cause the inactivation of the salivation signal through the sulfonation of either octopamine or dopamine. This, by itself, can cause RDS behaviors.

PANDAS is a subset of pediatric acute neuropsychiatric symptom (PANS). The current diagnosis of PANDAS and the hypothesized infection pathology is controversial [5]-[10]. Singer [10] [11] considered PANS to have numerous unknown causes, one of which might include hypodopaminergia [11]. The treatment of PANDAS remains disputed, especially since there have been limited efficacy studies to date [12]. Previously, we proposed an innovative potential treatment for PANDAS based on previous clinical trials using a pro-dopamine regulator known as KB220 variants. Our ongoing research suggests that achieving “dopamine homeostasis” by precision-guided DNA testing and pro-dopamine modulation could result in improved therapeutic outcomes [12].

It is indeed well known that PANDAS can result in a neuroinflammatory response influencing the dysfunction of TH1 and TH2 lymphocytes T cells. Importantly, these T cells are key mediators of adaptive immunity. Briefly, the specificity of the T cells response is related to the antigen-specific receptors they express, the **TCR**s (T Cell Receptor). It should be pointed out that, like immunoglobulins expressed by B cells, TCRs are somatically generated, and their diversity is obtained by random rearrangements of their gene segments. Moreover, T cells only recognize antigens once these have been processed into peptides and exposed onto the surface of the target cell, in an antigen presentation structure known as the **Major Histocompatibility Complex** (MHC). In fact, it is well known that the MHC-antigen complex binding to the TCR is the first mandatory step for T cell activation. Furthermore, the crucial role of Th1 and Th2 in the immune response, the mechanisms leading to the priming of naïve CD4+ T cells into Th1 or Th2, it is clear that the cytokine microenvironment is fundamental in the process, as well as the strength of the stimulatory signals from the APC (*i*.*e*., the affinity of TCR for the peptide ligand). Understanding this basic information provides the rationale whereby PANDAS and associated infection induce a disruption of this important physiological elemental construct [13]. While our study did not address this important issue, we are compelled to at least realize that in conjunction with our approach as described herein to offer potential assistance linked to the RDS type of behaviors, neuroinflammation must also be treated. For example, clinicians have employed a number of natural ingredients such as glutathione, perilla, cumin, luteolin, fisetin, and others known to reduce neuroinflammatory processes [14].

The present case report supports our hypothesis, serving as a starting rationale for conducting larger randomized-control studies on this topic.

## 2. Materials and Methods

### Sequence of Events:

The subject was a 12-year-old Caucasian male with no previous psychiatric episodes. On 7/20/2020, the patient tested for Genetic Addiction Risk Severity (GARS). GARS was initially conducted because MP (the mother), taking note of many unwanted behaviors (including suicidal ideation), knew of KB’s work in the area of RDS. The GARS results identified DNA polymorphisms associated with low dopamine function. While this test has been validated to capture polymorphisms of reward genes (see 17), the test has not as yet received approval from the FDA. The KB220 product, with customized ingredients matching the measured polymorphisms reflected in GARS results, was subsequently administered to induce “dopamine homeostasis” (KB220ZPBMPOLY [POLYGEN^®^]). The patient started the KB220z within 30 days of testing (8/18/2020).

**Figure 1.**
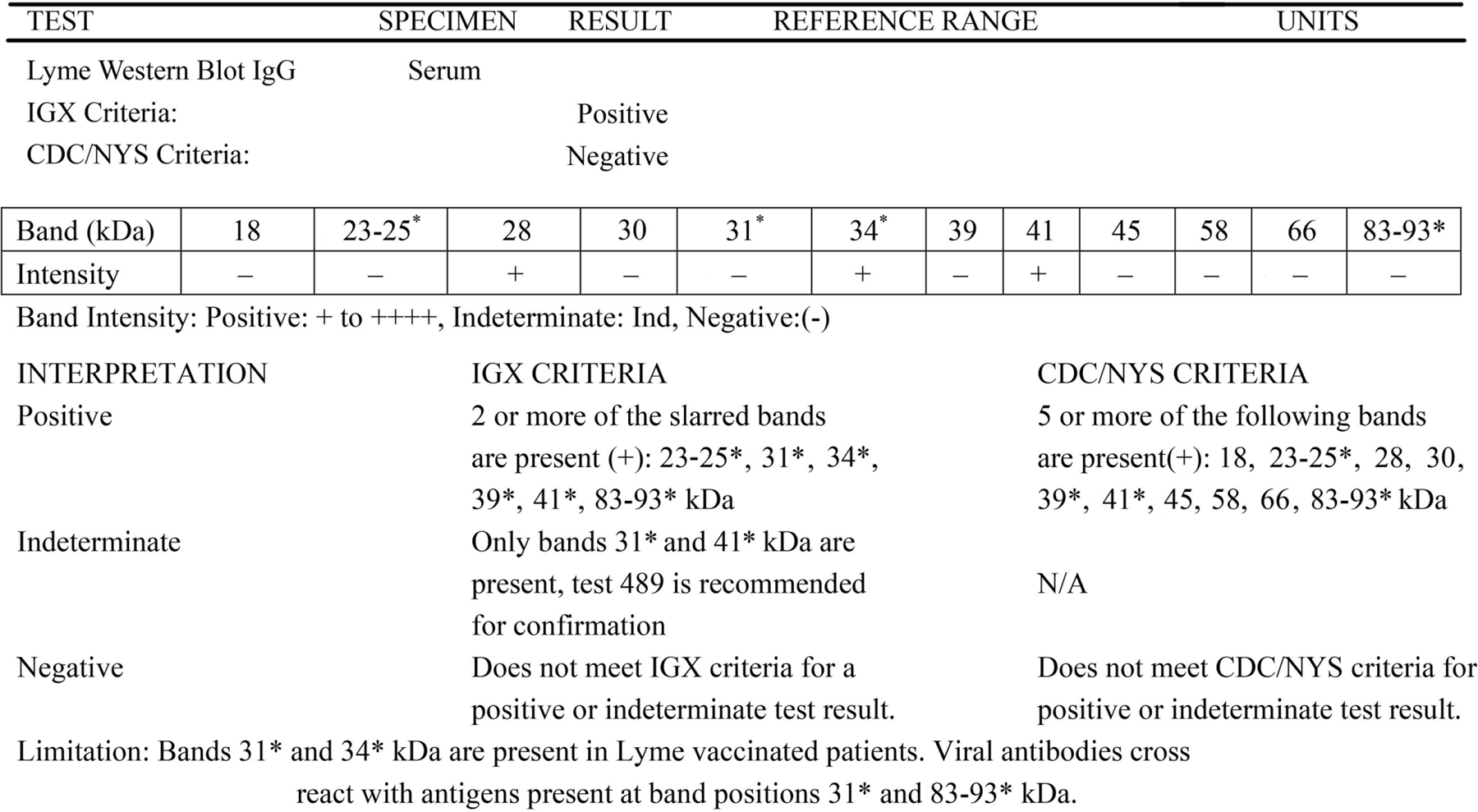
The results and interpretation of the patient’s Lyme Western Blot IgG. Results indicated that the patient was positive for Lyme, according to the IGX criteria. For a more comprehensive list of blood/antibody testing performed, please refer to Figure S1.

**Figure 2.**
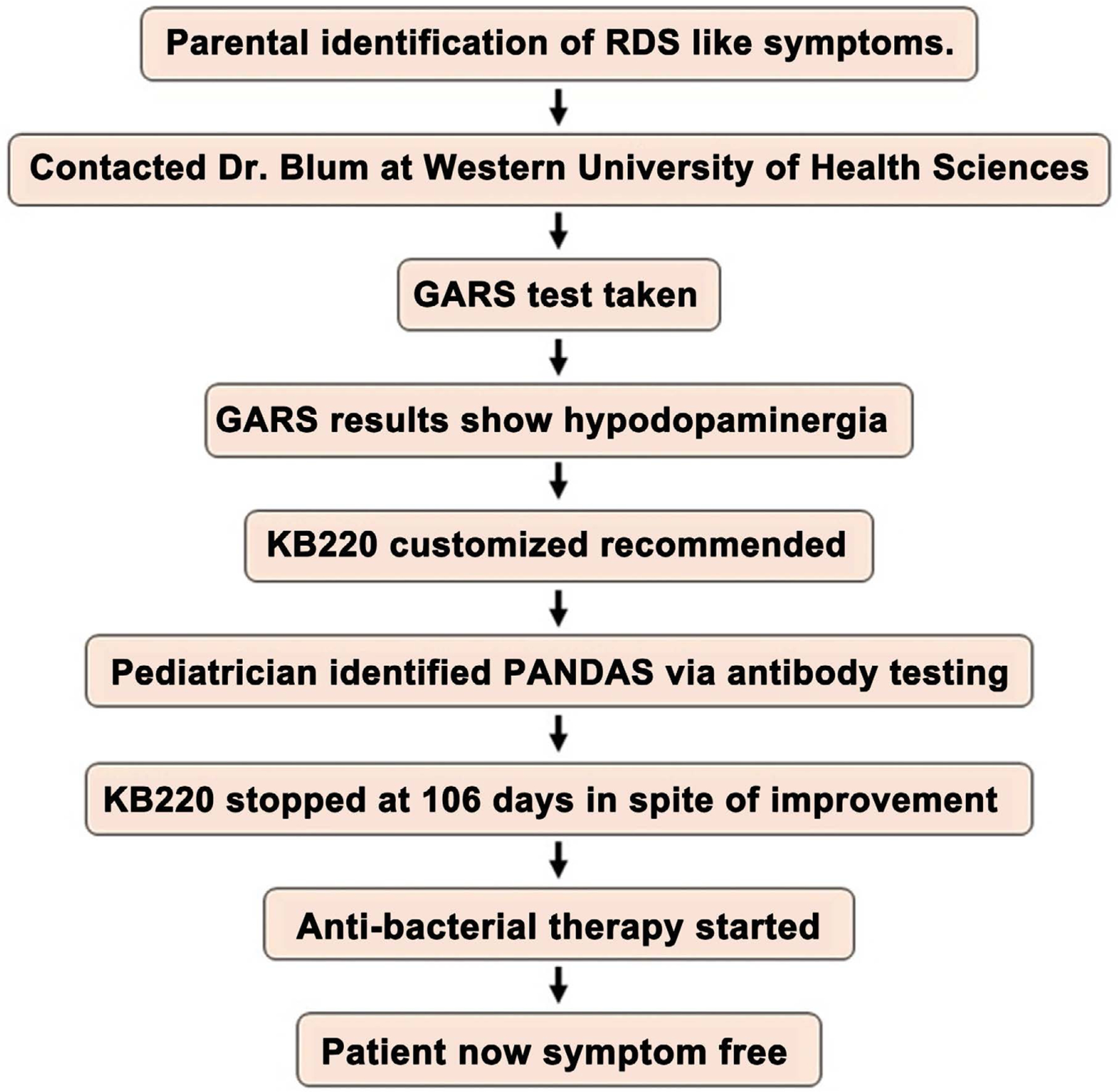
Flow chart summarizing the treatment events for the patient.

A physician suspected PANDAS/CANS following a visit on 2/18/2021. The pediatrician confirmed the diagnosis with antibody titers for Strep, Lyme, and Borrelia (Table 1, Figure 1 & Figure S1). Prior to this, neither MP nor KB considered PANDAS to be the reason for the rapid onset of RDS behaviors. The pro-dopamine regulator was administered for ***seven months*** prior to involving a pediatric PANDAS/CANS specialist. The patient was subsequently (2/19/21) placed on anti-bacterial medication, stopping the pro-dopamine regulator to ensure a reasonable wash-out period. This was done because the attending physician wanted to see the results of the antibacterial treatment alone. This sequence of events, which is unorthodox in terms of traditional patient care, is summarized in Figure 2.

### Genetic Testing:

In this case, study protocols were reviewed and approved by the PATH Foundation (NY) Institutional Review Boards (IRB). The genotyping data complied with accepted Genetic Information Non-Discrimination Act (GINA) and Health Insurance Portability and Accountability Act (HIPAA) practices, which are mandated by law to protect patient privacy. Written and informed consent was provided and approved by the primary participants.

The biotechnical methods used to identify GARS risk alleles have been previously published in detail [15]. Cheek swabs were obtained from the patient and taken to Genus Health (San Antonio, Texas) for further processing. PCR amplification was used to isolate DNA, which was then analyzed for polymorphisms in the following genes: DRD1, DRD2, DRD3, DRD4, OPRM1, COMT, DAT1, DRD4-R, GABRB3, HTTLPR, and MAOA. An index of the genes incorporated in the GARS assay and the specific risk polymorphisms are supplied in Tables 2(a)-(c).

KB220Z: The neuro-nutrients classified as KB220ZPAM/PBM have been genetically formulated to provide maximum results based on GARS results (Table 3). The six different formulations were scientifically developed based on a person’s genetic risk variants. Based on the patient’s GARS results, we used KB220ZPBMPOLY (POLYGEN), a genetically based formula created to provide precision supplementation to overcome dopamine, serotonin, endorphin, and GABA deficits. These deficits are the result of multiple systems and integrative polymorphic genes affecting the Brain Reward Cascade (BRC) and causing impairments across the BRC. This leads to low dopamine function, which in turn causes reward deficiency or an overall lack of well-being [16].

Putative Diagnostic Basis of PANDAS: Throat swabs from the patient underwent a rapid antigen detection test for Group A Streptococci, Lyme, and Borrelia, performed in accordance with the manufacturer’s instructions. Samples were placed into a tube with a standard reagent solution and mixed forcefully in order to extract the specimen into the suspended solution. After mixing and then subsequently letting the solution stand for two minutes, the swab was removed and discarded. The absorbent end of a test stick was then placed into the tube with the extracted specimen. Results were read after 5 minutes. The results were considered positive if a test line and control line appeared on the test stick and negative if only the control line appeared. A positive result indicated that the assay had detected Group A Streptococcus antigen within the extracted specimen. A negative result indicated that there was no Group A Streptococcus antigen within the extracted specimen.

Based on the positive diagnostic workup by the attending physician utilizing standard antibody testing, the presence of antibodies to Streptococcus, and Lyme but not Borrelia were found. The patient was placed on the following antibacterial medications: Amoxicillin-Clavulanic acid, 875 – 125 mg (Augmentin); Sulfamethoxazole-trimethoprim 800 – 160 mg (Bactrim); Naproxen 500 mg (Naproxen); Doxycycline Hyclate, 100 mg; Cefdinir, 300 mg. (All BID, generally tolerated, some stomach issues presented).

### Statistical Analysis

**Figure 3.**
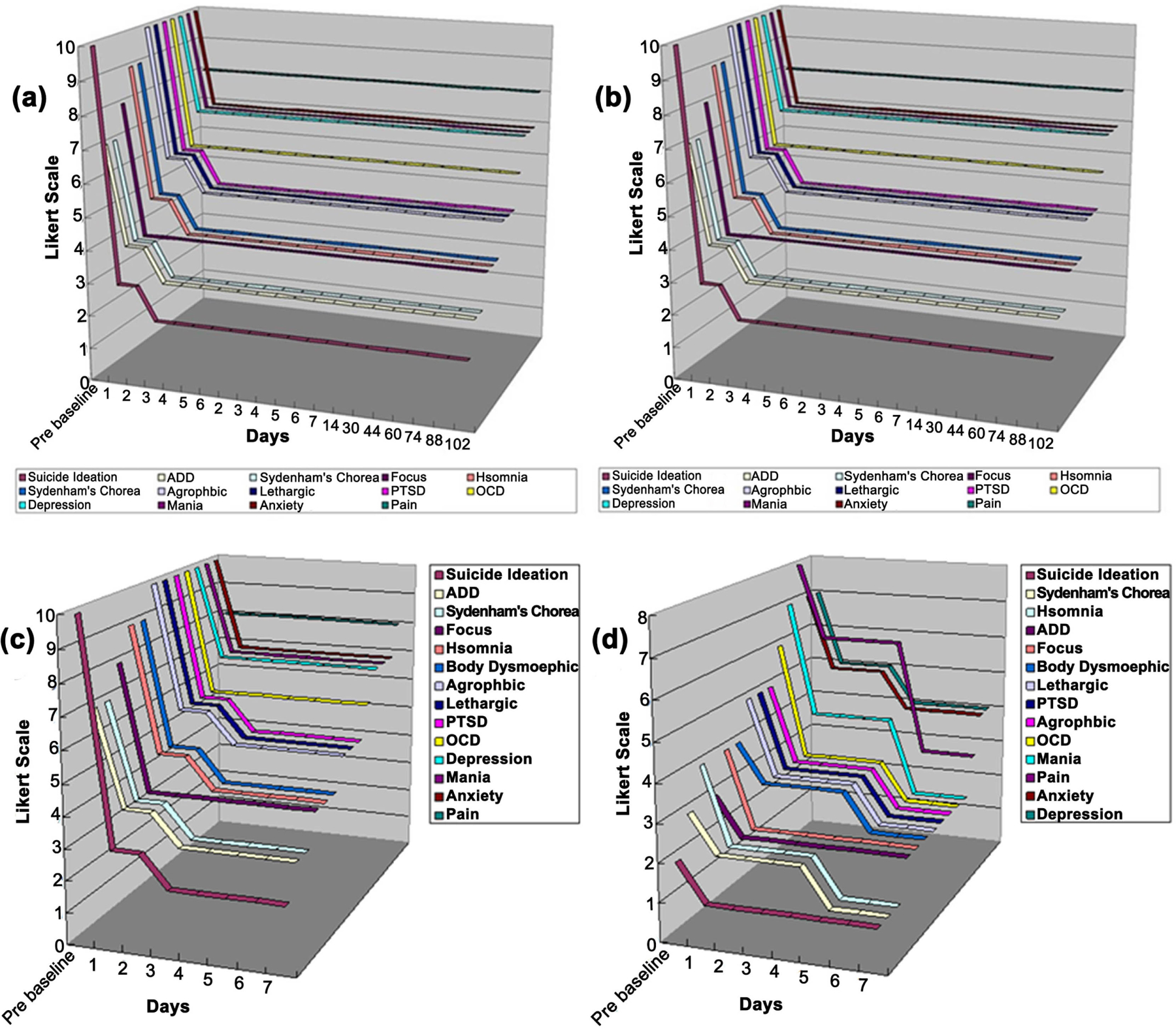
(a) shows the pro-dopamine regulation pre- and post-behavioral symptoms. (b) shows the antibacterial pre- and postbehavioral symptoms. (c) shows the first seven consecutive days for the pro-dopamine regulation pre- and post-behavioral symptoms. (d) shows the first seven consecutive days for the antibacterial pre- and post-behavioral symptoms.

A monotonic trend analysis was performed on each outcome measure (in Likert scales), showing a consistently decreasing trend when compared to the baseline measurement (p < 0.05). The data was tabulated by the mother (MP) as self-reported by the patient (AP) and further verified by the father. Because of potential bias, all three had to agree with the data entry. The patient’s tabulated behavioral response, including behavioral symptoms (pain, suicide ideation, focus, etc.) and psychiatric syndromes (agoraphobic, body-dysmorphic, etc.) have been graphed in Figure 3(a) (pro-dopamine regulation) and Figure 3(b) (antibacterial). While a psychiatrist was not involved in these entries, we feel confident that the described responses are correct.

## 3. Results

GARS test results revealed four heterozygous polymorphic risk alleles: DRD2/ ANKK (Taq1A), OPRM1 (A/G), DRD3 (C/T), and MAOA (4R), revealing hypo-dopaminergia. Based on these results, the patient was immediately placed on KB220ZPBMPOLY. The patient’s tabulated behavioral results are found in Figure 3 and Table S1. Based on the data in Figure 3, all features were found to decrease over time when compared to the baseline measurements, except for pain (no change was observed). There was very rapid relief of many symptoms in the patient from day one, and that was maintained for 102 days. There was no significant trend due to this surprising rapid relief finding.

In the second (antibacterial) phase of treatment, all features decreased in scale. Suicide Ideation, OCD, and Focus were insignificant (p > 0.14). Depression (p = 0.04), Anxiety (p = 0.04), and Pain (p = 0.022) significantly decreased over time. Mania (p = 0.08), Sydenham’s Chorea (p = 0.06), Acrophobia (p = 0.1), Insomnia (p = 0.1), Body Dysmorphic Disorder (p = 0.06), Lethargy (p = 0.1), and PTSD (p = 0.1) marginally decreased, with significant decreases found in the first five days; Mania (p = 0.039), Sydenham’s Chorea (p = 0.034), Acrophobic (p = 0.048), Insomnia (p = 0.048), Body Dysmorphic Disorder (p = 0.034), Lethargic (p = 0.048), and PTSD (p = 0.048) (Figure 3 and Table S2).

Abnormal result values in Table 1 are bolded. Results were positive for DNase antibody, Mycoplasma pneumoniae antibody IgG and IgM, EBV viral capsid AG, and EBV nuclear AG. Additionally, results revealed a vitamin D insufficiency and herpesvirus 6 antibody IgG, suggesting previous infection. While the titer of Strep at < 50 IUm appeared to be related to an earlier infection rather than active, the pediatrician diagnosed the patient’s condition as PANDAS. A comprehensive list of blood/antibody testing is found in Figure S1.

## 4. Discussion

This case study is the first to test GARS with precision matched KB220 pro-dopamine regulation as a frontline treatment of PANDAS/CANS in a hypodopaminergic comprised adolescent with polymorphic dopamine D2 (-DRD2/ANKK (Taq1A)), OPRM1 (A/G), DRD3 (C/T), and MAOA (4R) risk alleles. These polymorphic risk alleles impact many RDS behaviors [17] presently observed in this patient. The innovative GARS system can successfully stratify the risk for developing RDS, which can be helpful for individuals in recovery or, in the present case, for those with full-blown RDS behaviors [15] [17].

Extreme RDS-like behaviors can co-occur with Strep and Lyme infection, especially in young people with DNA-induced hypodopaminergia [18]. Furthermore, there is limited treatment and unbiased research available for PANDAS and CANS [19]. Antibacterial therapy for PANDAS has yielded some success, yet there is no such treatment for CANS since it is not the result of bacterial insult. Moreover, antibacterial therapies only provide a modest relief of symptoms over time. In spite of the diagnosis of PANDAS by the attending pediatrician and the positive response to antibacterial therapy, it is indeed possible that this patient’s case could be classified as CANS. Since the titer for Streptococcal was below the active infection level, while Lyme was at an active titer level, while not certain, the pediatrician’s diagnosis seems prudent.

Indeed, another caveat relates to the use and even further development of the GARS as a novel methodology to deliver customized or even semi-customized KB220. While it is true that there are common variants in the general population, it is also true that those who carry RDS have a significantly higher prevalence of at least eight out of ten variants, as measured by GARS [20]. DNA customization is important with the use of KB220, and most people with RDS (87%) require the Polygen formula of KB220 described above.

Nevertheless, if our findings hold up with the required double-blinded studies and an increased number of cases, certainly in terms of clinical practice, a quicker relief that could be maintained early on (dopamine homeostasis [21]) seems most prudent. Our recommendation, as such, is to utilize a combined therapeutic approach. The frontline utilization of GARS coupled with precision pro-dopamine KB220 variants following antibody diagnosis of bacterial infection (Strep/Lyme) may become a real futuristic option following more investigation. It should also be coupled with products containing glutathione, perilla, cumin, luteolin, fisetin, and others known to reduce neuroinflammatory processes.

In summary, treatment of neuropsychiatric symptoms with precision-guided DNA testing and pro-dopamine regulation in a genetically hypodopaminergic preadolescent male with putative PANDAS/CANS yielded a positive clinical outcome, suggesting epigenetic repair. It is noteworthy that one of us (DE) found similar benefits in treating teens diagnosed with PANDAS with precision KB220 variants, which will be the subject of a subsequent follow-up article [22]-[32].

## 5. Limitations

This study is limited by sample size and a non-conventional treatment approach. A more extensive controlled study is required to confirm the significance of these findings. An inherent bias in the self-reported data by the three-way agreement of the family members must be considered. Furthermore, the diagnosis of PANDAS needs to be questioned since the streptococcal titer was below the required active level in spite of a positive titer for Lyme. It is also possible that the time differential between first utilizing the KB220 variant and the putative diagnosis of PANDAS, was reflected by the presence of antibodies to previous streptococcal infection, which might explain the improvement with antibacterial therapy (see results). One might also consider the possibility of testing plasma dopamine levels pre and post treatment especially with the Pro-dopamine regulation regime as administered in this case. However, it is difficult to distinguish peripheral vs brain dopamine in plasma levels. The most reliable way to test brain dopamine levels is via intrathecal testing [33]. This latter methodology is very difficult, especially for our youth.

We suggest herein that in the future, larger population RTC’s should utilize GARS testing coupled with mRNA profiling, as we published previously [34]. We are also cognizant of racial genetic polymorphic differences, and this must also be taken into consideration [35]. Interestingly, even if we can’t ensure a clear diagnosis of PANDAS perse, a diagnosis of CANS seems parsimonious. We incorporated the term “epigenetic” to infer environmentally induced manipulation of the core neurotransmitter signaling, but in the present study, we have not actually shown any effect on histone post-translational alteration in terms of methylation conversion to acetylation, the subject of another experiment.

The GARS test, in general, assesses the possibility of benefit from medication-assisted treatment with dopamine amplification. RDS methodology possesses a strong promise for diminishing the burden of hypodopaminergia by guiding the restoration of dopamine homeostasis, which could also affect any circadian rhythm insults [36]-[38].

While these initial results are encouraging, of course, more research is required, especially double-blinded placebo-controlled studies comparing antibacterial therapy alone, KB220 therapy alone [36], and coupling of the two modalities. Until these studies are completed, we must be cautious regarding any clinically relevant interpretation of these results [39].

Moreover, in the method section, we specified that a p-value of less than 5% was considered statistically significant for each test. However, regarding the FWER (family-wise error rate) control, we have not applied the Bonferroni correction since it is known as unsuitable for pilot/exploratory studies, which are intended to generate hypotheses and not confirm them.

## 6. Conclusion

Our recommendation, albeit possibly biased, is to utilize and further research a combined therapeutic approach, involving precision-guided DNA testing, prodopamine regulation along with antibacterial therapy, and anti-neuroinflammation agents in patients with suspected PANDAS/CANS.

## Supplementary Material

1

## Figures and Tables

**Table 1.** Pertinent blood testing/antibody titer results.

DNASE B ANTIBODY	622 (U/mL)	<376 Negative	
VITAMIN D, 25-OH, TOTAL, IA	25 (ng/mL)	Deficiency: <20 ng/mL	
		Insufficiency: 20 – 29 ng/mL	
		Optimal: > or = 30 ng/mL	
MYCOPLASMA PNEUMONIAE ANTIBODIES (IGG, IGM)			
MYCOPLASMA PNEUMONIAE ANTIBODY (IGG)	1.71	≤0.90	Negative
		0.91 – 1.09	Equivocal
		≥1.10	Positive

MYCOPLASMA PNEUMONIAE ANTIBODY (IGM)	671 (U/mL)	<770 U/ml	Negative
		770 – 950 U/mL	Low positive
		>950 U/mL	Positive
EPSTEIN BARR VIRUS ANTIBODY PANEL			
EBV VIRAL CAPSID AG (VCA)	<36.00 (U/mL)	<36.00	Negative
		36.00 – 43.99	Equivocal
		>43.99	Positive

EBV VIRAL CAPSID AG (VCA)	220.00 (U/mL)	<18.00	Negative
		18.00 – 21.99	Equivocal
		>21.99	Positive

EBV NUCLEAR AG (EBNA)	>600.00 (U/mL)	<18.00	Negative
		18.00 – 21.99	Equivocal
		>21.99	Positive
HERPESVIRUS 6 ANTIBODIES (IGG, IGM)			
HERPESVIRUS 6 AB (IGG)	1:10 (titer)	<1:10 Negative	
HERPESVIRUS 6 AB (IGM)	<1:20 (titer)	<1:20 Negative	

**Table 2.** (a) single nucleotide polymorphisms (SNPs); (b) simple sequence of repeats (variable number tandem repeats and insertion/deletions); (c) dinucleotide repeats.

(a)			

GENE	POLYMORPHISM	VARIANT ALLELES	RISK ALLELE
Dopamine D1 Receptor DRD1	rs4532	A/G	A
Dopamine D2 Receptor DRD2	rs1800497	A/G (A1/A2)	A (A1)
Dopamine D3 Receptor DRD3	rs6280	C/T	C
Dopamine D4 Receptor DRD4	rs1800955	C/T	C
Catechol-O-Methyltransferase COMT	rs4680	A/G (Met/Val)	G (Val)
Mu-Opioid Receptor OPRM1	rs1799971	A/G (As/Asp)	G (Asp)
(b)			
GENE	POLYMORPHISM	VARIANT ALLELES	RISK ALLELE

Dopamine D4 Receptor DRD4	rs761010487	48bp repeat 2R - 11R	≥7R, long form
Dopamine Active Transporter DAT1	rs28363170	40p repeat 3R - 11R	< 9R
Monoamine Oxidase A MAOA	rs768062321	30bp repeat 2R - 5R	3.5R, 4R, 5R
Serotonin Transporter SLC6A4 (5-HTTLPR)	rs4795541, rs25531	43bp repeat, with SNP L/XL and S, G/A	S, LG
(c)			
GENE	POLYMORPHISM	VARIANT ALLELES	RISK ALLELE

GABA(A) Receptor, Alpha-3 GABRB3	rs764926719	CA dinucleotide repeat 171 – 201bp sized fragments	181

**Table 3.** GARS repeats primer details.

PRIMER	SEQUENCE (5’ to 3’)	5’ LABEL	REACTION (nM)
AMELO-F	CCC TGG GCT CTG TAA AGA ATA GTG	NED	150
AMELO-R	ATC AGA GCT TAA ACT GGG AAG CTG	‒	
MAO-F	ACA GCC TGA CCG TGG AGA AG	NED	120
MAO-R	GAA CGG ACG CTC CAT TCG GA	‒	
DAT-F	TGT GGT GTA GGG AAC GGC CTG AG	6FAM	120
DAT-R	CTT CCT GGA GGT CAC GGC TCA AGG	‒	
DRD4-F	GCT CAT GCT GCT GCT CTA CTG GGC	VIC	480
DRD4-R	CTG CGG GTC TGC GGT GGA GTC TGG	‒	
GABRA-F	CTC TTG TTC CTG TTG CTT TCA ATA CAC	NED	120
GABRA-R	CAC TGT GCT AGT AGA TTC AGC TC	‒	
HTTLPR-F	ATG CCA GCA CCT AAC CCC TAA TGT	PET	120
HTTLPR-R	GAG GGA CTG AGC TGG ACA ACC AC	‒	

## References

[1] Moretti G, Pasquini M, Mandarelli G, Tarsitani L and Biondi M (2008) What Every Psychiatrist Should Know about PANDAS: A Review. Clinical Practice and Epidemiology in Mental Health, 4, Article No. 13. 10.1186/1745-0179-4-13 PMC2413218 18495013

[2] Fallon BA, Madsen T, Erlangsen A and Benros ME (2021) Lyme Borreliosis and Associations with Mental Disorders and Suicidal Behavior: A Nationwide Danish Cohort Study. American Journal of Psychiatry, 178, 921–931. 10.1176/appi.ajp.2021.20091347 34315282

[3] Madigan M, Gupta A, Bowirrat A, Baron D, Badgaiyan R, Elman I, (2022) Precision Behavioral Management (PBM) and Cognitive Control as a Potential Therapeutic and Prophylactic Modality for Reward Deficiency Syndrome (RDS): Is There Enough Evidence? International Journal of Environmental Research and Public Health, 19, Article 6395. 10.3390/ijerph19116395 PMC9180535 35681980

[4] Blum K, Modestino EJ, Febo M, Steinberg B, McLaughlin T, Fried L, (2017) Lyme and Dopaminergic Function: Hypothesizing Reduced Reward Deficiency Symptomatology by Regulating Dopamine Transmission. Journal of Systems and Integrative Neuroscience, 3, 1–4. 10.15761/jsin.1000163 PMC5521197 28736624

[5] Pichichero ME (2008) The PANDAS Syndrome. In: Finn A, Curtis N and Pollard A, Eds., Hot Topics in Infection and Immunity in Children V, Springer, 205–216. 10.1007/978-0-387-79838-7_17

[6] Marazziti D, Mucci F and Fontenelle LF (2018) Immune System and Obsessive-Compulsive Disorder. Psychoneuroendocrinology, 93, 39–44. 10.1016/j.psyneuen.2018.04.013 29689421

[7] Leckman JF, Denys D, Simpson HB, Mataix-Cols D, Hollander E, Saxena S, (2010) Obsessive-Compulsive Disorder: A Review of the Diagnostic Criteria and Possible Subtypes and Dimensional Specifiers for DSM-V. Depression and Anxiety, 27, 507–527. 10.1002/da.20669 20217853 PMC3974619

[8] Kurlan R and Kaplan EL (2004) The Pediatric Autoimmune Neuropsychiatric Disorders Associated with Streptococcal Infection (PANDAS) Etiology for Tics and Obsessive-Compulsive Symptoms: Hypothesis or Entity? Practical Considerations for the Clinician. Pediatrics, 113, 883–886. 10.1542/peds.113.4.883 15060240

[9] Shprecher D and Kurlan R (2009) The Management of Tics. Movement Disorders, 24, 15–24. 10.1002/mds.22378 19170198 PMC2701289

[10] Singer HS (2011) Tourette Syndrome and Other Tic Disorders. Handbook of Clinical Neurology, 11, 641–657. 10.1016/b978-0-444-52014-2.00046-x 21496613

[11] Singer HS, Gilbert DL, Wolf DS, Mink JW and Kurlan R (2012) Moving from PANDAS to Cans. The Journal of Pediatrics, 160, 725–731. 10.1016/j.jpeds.2011.11.040 22197466

[12] Blum K, Thanos PK, Badgaiyan RD, Febo M, Oscar-Berman M, Fratantonio J, Demetrovics Z and Gold MS (2022) Neurogenetics and Gene Therapy for Reward Deficiency Syndrome: Are We Going to the Promised Land? Expert Opinion on Biological Therapy, 15, 973–985. 10.1517/14712598.2015.1045871 25974314

[13] Kulumani Mahadevan LS, Murphy M, Selenica M, Latimer E and Harris BT (2023) Clinicopathologic Characteristics of PANDAS in a Young Adult: A Case Report. Developmental Neuroscience, 45, 335–341. 10.1159/000534061 37699369 PMC10753865

[14] Xie K, Chai Y, Lin S, Xu F and Wang C (2021) Luteolin Regulates the Differentiation of Regulatory T Cells and Activates IL-10-Dependent Macrophage Polarization against Acute Lung Injury. Journal of Immunology Research, 2021, Article 8883962. 10.1155/2021/8883962 PMC7834791 33532509

[15] Blum K, Bowirrat A, Baron D, Lott L, Ponce JV, Brewer R, (2020) Bio-technical Development of Genetic Addiction Risk Score (GARS) and Selective Evidence for Inclusion of Polymorphic Allelic Risk in Substance Use Disorder (SUD). Journal of Systems and Integrative Neuroscience, 6, 1–20. 10.15761/jsin.1000221 PMC7891477 33614164

[16] Blum K, Ashford JW, Kateb B, Sipple D, Braverman E, Dennen CA, (2012) Dopaminergic Dysfunction: Role for Genetic & Epigenetic Testing in the New Psychiatry. Journal of the Neurological Sciences, 453, Article 120809. 10.1016/j.jns.2023.120809 37774561

[17] Blum K, Kazmi S, Modestino EJ, Downs BW, Bagchi D, Baron D, (2021) A Novel Precision Approach to Overcome the “Addiction Pandemic” by Incorporating Genetic Addiction Risk Severity (GARS) and Dopamine Homeostasis Restoration. Journal of Personalized Medicine, 11, Article 212. 10.3390/jpm11030212 PMC8002215 33809702

[18] Blum K (2016) Hypothesizing that a Pro-Dopaminergic Regulator (KB220z^™^ Liquid Variant) Can Induce “Dopamine Homeostasis” and Provide Adjunctive Detoxification Benefits in Opiate/Opioid Dependence. Clinical Medical Reviews and Case Reports, 3, Article 125. 10.23937/2378-3656/1410125 PMC5638455 29034323

[19] Sigra S, Hesselmark E and Bejerot S (2018) Treatment of PANDAS and PANS: A Systematic Review. Neuroscience & Biobehavioral Reviews, 86, 51–65. 10.1016/j.neubiorev.2018.01.001 29309797

[20] Blum K, Han D, Gupta A, Baron D, Braverman ER, Dennen CA, (2022) Statistical Validation of Risk Alleles in Genetic Addiction Risk Severity (GARS) Test: Early Identification of Risk for Alcohol Use Disorder (AUD) in 74,566 Case-Control Subjects. Journal of Personalized Medicine, 12, Article 1385. 10.3390/jpm12091385 PMC9505592 36143170

[21] Blum K (2017) Dopamine Homeostasis Brain Functional Connectivity in Reward Deficiency Syndrome. Frontiers in Bioscience, 22, 669–691. 10.2741/4509 27814639

[22] Blum K, Dennen CA, Braverman ER, Gupta A, Baron D, Downs BW, (2022) Hypothesizing that Pediatric Autoimmune Neuropsychiatric Associated Streptococcal (PANDAS) Causes Rapid Onset of Reward Deficiency Syndrome (RDS) Behaviors and May Require Induction of “Dopamine Homeostasis”. Open Journal of Immunology, 12, 65–75. 10.4236/oji.2022.123004 36407790 PMC9670240

[23] Endres D, Pollak TA, Bechter K, Denzel D, Pitsch K, Nickel K, (2022) Immunological Causes of Obsessive-Compulsive Disorder: Is It Time for the Concept of an “Autoimmune OCD” Subtype? Translational Psychiatry, 12, Article No. 5. 10.1038/s41398-021-01700-4 PMC8744027 35013105

[24] Cocuzza S, Maniaci A, La Mantia I, Nocera F, Caruso D, Caruso S, (2022) Obsessive-Compulsive Disorder in PANS/PANDAS in Children: In Search of a Qualified Treatment—A Systematic Review and Metanalysis. Children, 9, Article 155. 10.3390/children9020155 PMC8869780 35204876

[25] Hutanu A, Reddy LN, Mathew J, Avanthika C, Jhaveri S and Tummala N (2022) Pediatric Autoimmune Neuropsychiatric Disorders Associated with Group a Streptococci: Etiopathology and Diagnostic Challenges. Cureus, 14, e27729. 10.7759/cureus.27729 36106298 PMC9447625

[26] Trifonova EA, Mustafin ZS, Lashin SA and Kochetov AV (2022) Abnormal mTOR Activity in Pediatric Autoimmune Neuropsychiatric and MIA-Associated Autism Spectrum Disorders. International Journal of Molecular Sciences, 23, Article 967. 10.3390/ijms23020967 PMC8781199 35055151

[27] O’Dor SL, Zagaroli J, Belisle R, Hamel M, Downer O, Homayoun S, (2022) The COVID-19 Pandemic and Children with PANS/PANDAS: An Evaluation of Symptom Severity, Telehealth, and Vaccination Hesitancy. Child Psychiatry & Human Development, 55, 327–335. 10.1007/s10578-022-01401-z 35930178 PMC9361990

[28] Prus K, Weidner K and Alquist C (2022) Therapeutic Plasma Exchange in Adolescent and Adult Patients with Autoimmune Neuropsychiatric Disorders Associated with Streptococcal Infections. Journal of Clinical Apheresis, 37, 597–599. 10.1002/jca.22023 36251457 PMC10092170

[29] Aman M, Coelho JS, Lin B, Lu C, Westwell-Roper C, Best JR, (2022) Prevalence of Pediatric Acute-Onset Neuropsychiatric Syndrome (PANS) in Children and Adolescents with Eating Disorders. Journal of Eating Disorders, 10, Article No. 194. 10.1186/s40337-022-00707-6 PMC9749211 36514161

[30] Ellerkamp H, Thienemann M, Tinero J, Shaw R, Dowtin LL, Frankovich J, (2022) Group Psychotherapy for Parents of Youth with Pediatric Acute-Onset Neuropsychiatric Syndrome. Journal of Clinical Psychology in Medical Settings, 30, 660–672. 10.1007/s10880-022-09926-0 36480109

[31] Gromark C, Hesselmark E, Djupedal IG, Silverberg M, Horne A, Harris RA, (2021) A Two-to-Five Year Follow-Up of a Pediatric Acute-Onset Neuropsychiatric Syndrome Cohort. Child Psychiatry & Human Development, 53, 354–364. 10.1007/s10578-021-01135-4 33559023 PMC7870456

[32] Efe A (2022) SARS-CoV-2/COVID-19 Associated Pediatric Acute-Onset Neuropsychiatric Syndrome a Case Report of Female Twin Adolescents. Psychiatry Research Case Reports, 1, Article 100074. 10.1016/j.psycr.2022.100074 PMC9562621 36267397

[33] Blum K, Oscar-Berman M, Stuller E, Miller D, Giordano J, Morse S, (2012) Neurogenetics and Nutrigenomics of Neuro-Nutrient Therapy for Reward Deficiency Syndrome (RDS): Clinical Ramifications as a Function of Molecular Neurobiological Mechanisms. Journal of Addiction Research & Therapy, 3, Article 139. 10.4172/2155-6105.1000139 PMC3733258 23926462

[34] Blum K, Steinberg B, Gondré-Lewis MC, Baron D, Modestino EJ, Badgaiyan RD, (2021) A Review of DNA Risk Alleles to Determine Epigenetic Repair of Mrna Expression to Prove Therapeutic Effectiveness in Reward Deficiency Syndrome (RDS): Embracing “Precision Behavioral Management”. Psychology Research and Behavior Management, 14, 2115–2134. 10.2147/prbm.s292958 34949945 PMC8691196

[35] Abijo T, Blum K and Gondré-Lewis MC (2020) Neuropharmacological and Neurogenetic Correlates of Opioid Use Disorder (OUD) as a Function of Ethnicity: Relevance to Precision Addiction Medicine. Current Neuropharmacology, 18, 578–595. 10.2174/1570159×17666191118125702 31744450 PMC7457418

[36] Blum K, McLaughlin T, Bowirrat A, Modestino EJ, Baron D, Gomez LL, (2022) Reward Deficiency Syndrome (RDS) Surprisingly Is Evolutionary and Found Everywhere: Is It “Blowin’ in the Wind”? Journal of Personalized Medicine, 12, Article 321. 10.3390/jpm12020321 PMC8875142 35207809

[37] Blum K, Sheridan PJ, Wood RC, Braverman ER, Chen TJH, (1996) The D2 Dopamine Receptor Gene as a Determinant of Reward Deficiency Syndrome. Journal of the Royal Society of Medicine, 89, 396–400. 10.1177/014107689608900711 8774539 PMC1295855

[38] Blum K, Modestino EJ, Gondre-Lewis MC, Baron D, Steinberg B, Thanos PK, (2018) Pro-Dopamine Regulator (KB220) A Fifty Year Sojourn to Combat Reward Deficiency Syndrome (RDS): Evidence Based Bibliography (Annotated). CPQ Neurology and Psychology, 1. https://www.cientperiodique.com/journal/fulltext/CPQNP/1/2/13 PMC6448775 30957097

[39] Blum K, Baron D, McLaughlin T, Thanos PK, Dennen C, Ceccanti M, (2022) Summary Document Research on RDS Anti-Addiction Modeling: Annotated Bibliography. Journal of Systems and Integrative Neuroscience, 8, 2–35. PMC11100022 38765881

