# Supplementary material for: The First Pilot Epigenetic Type Improvement of Neuropsychiatric Symptoms in a Polymorphic Dopamine D2 (-DRD2/ANKK (Taq1A)), OPRM1 (A/G), DRD3 (C/T), and MAOA (4R) Compromised Preadolescence Male with Putative PANDAS/CANS: Positive Clinical Outcome with Precision-Guided DNA Testing and Pro-Dopamine Regulation (KB220) and Antibacterial Therapies
Source: Open J Immunol. Author manuscript; Available in PMC 2024 Nov 6. (PMC11539193; doi:10.4236/oji.2024.143006)
Supplement: 1 [file NIHMS2026239-supplement-1.pdf]

Supplemental Figures

Accession #: [redacted] **Client Info.** [redacted]
Lab: [redacted]
Patient: [redacted]
Pat. Chart No.: [redacted] [redacted] [redacted] [redacted]
Ordering Phys: [redacted]

Lab Order No.: [redacted] **Collected:** [redacted]
Print Date: [redacted] **Received:** [redacted]
Report Status: [redacted] **Reported:** [redacted]

Test Name	Flag	Result Value (Unit)	Reference Range	Lab
**CBC (INCLUDES DIFF/PLT)**				
WHITE BLOOD CELL COUNT		6.8 (Thousand/uL)	4.5-13.5	NL1
RED BLOOD CELL COUNT		5.09 (Million/uL)	4.00-5.20	NL1
HEMOGLOBIN		13.6 (g/dL)	11.5-15.5	NL1
HEMATOCRIT		40.2 (%)	35.0-45.0	NL1
MCV		79.0 (fL)	77.0-95.0	NL1
MCH		26.7 (pg)	25.0-33.0	NL1
MCHC		33.8 (g/dL)	31.0-36.0	NL1
RDW		13.1 (%)	11.0-15.0	NL1
PLATELET COUNT		267 (Thousand/uL)	140-400	NL1
MPV		11.1 (fL)	7.5-12.5	NL1
ABSOLUTE NEUTROPHILS		4277 (cells/uL)	1500-8000	NL1
ABSOLUTE LYMPHOCYTES		1822 (cells/uL)	1500-6500	NL1
ABSOLUTE MONOCYTES		571 (cells/uL)	200-900	NL1
ABSOLUTE EOSINOPHILS		82 (cells/uL)	15-500	NL1
ABSOLUTE BASOPHILS		48 (cells/uL)	0-200	NL1
NEUTROPHILS		62.9 (%)		NL1
LYMPHOCYTES		26.8 (%)		NL1
MONOCYTES		8.4 (%)		NL1
EOSINOPHILS		1.2 (%)		NL1
BASOPHILS		0.7 (%)		NL1
**Comprehensive Metabolic Panel**				
Glucose		89 (mg/dL)	65-99	NL1
**Note:** Fasting reference interval				
Urea Nitrogen (bun)		13 (mg/dL)	7-20	NL1
Creatinine		0.47 (mg/dL)	0.30-0.78	NL1
**Note:** Patient is <18 years old. Unable to calculate eGFR.				
Bun/creatinine Ratio		NOT APPLICABLE ((calc))	6-22	NL1
Sodium		139 (mmol/L)	135-146	NL1
Potassium		4.0 (mmol/L)	3.8-5.1	NL1
Chloride		103 (mmol/L)	98-110	NL1
Carbon Dioxide		25 (mmol/L)	20-32	NL1
Calcium		10.0 (mg/dL)	8.9-10.4	NL1
Protein, Total		7.0 (g/dL)	6.3-8.2	NL1
Albumin		4.6 (g/dL)	3.6-5.1	NL1
Globulin		2.4 (g/dL (calc))	2.1-3.5	NL1
Albumin/globulin Ratio		1.9 ((calc))	1.0-2.5	NL1
Bilirubin, Total		0.4 (mg/dL)	0.2-1.1	NL1
Alkaline Phosphatase		244 (U/L)	123-426	NL1
Ast		24 (U/L)	12-32	NL1

[redacted]

Page 1 continued on 2

Accession #: [redacted] **Client Info.** [redacted]
Lab: [redacted]
Patient: [redacted]
Pat. Chart No.: [redacted]
Ordering Phys: [redacted]

Alt		20 (U/L)	8-30	NL1
**ANTI-STREPTOLYSIN O**				
ANTI-STREPTOLYSIN O		<50 (IU/mL)	<250	NL1
**MYCOPLASMA PNEUMONIAE ANTIBODIES (IGG,IGM)**				
Mycoplasma Pneumoniae Antibody (igg)	H	1.71	<=0.90	AMD

Note:
Reference Range:
<=0.90 Negative
0.91-1.09 Equivocal
>=1.10 Positive

A positive IgG result indicates that the patient has antibody to Mycoplasma. It does not differentiate between an active or past infection. The clinical diagnosis must be interpreted in conjunction with the clinical signs and symptoms of the patient.

Mycoplasma Pneumoniae Antibody (igm)		671 (U/mL)	<770	AMD

Note:
Reference Range:
<770 U/ml Negative
770-950 U/mL Low positive
>950 U/mL Positive

A positive IgM antibody result is consistent with recent infection. However, a negative result does not necessarily rule out recent infection as some individuals may not mount another IgM response, if previously infected.

A positive IgM antibody result with or without a positive IgG antibody result, is consistent with recent infection. However, a negative result does not necessarily rule out recent infection as some individuals may not mount another IgM response, if previously infected. A positive IgG antibody result in the absence of a positive IgM antibody result, indicates that the patient has antibody to Mycoplasma. It does not differentiate between an active or past infection. The clinical diagnosis must be interpreted in conjunction with the clinical signs and symptoms of the patient.

**DNASE B ANTIBODY**				
DNASE B ANTIBODY	H	622 (U/mL)	<376	SLI
**HOMOCYSTEINE**				
HOMOCYSTEINE		5.3 (umol/L)	<11.4	NL1

Note: Homocysteine is increased by functional deficiency of folate or vitamin B12. Testing for methylmalonic acid differentiates between these deficiencies. Other causes of increased homocysteine include renal failure, folate antagonists such as methotrexate and phenytoin, and exposure to nitrous oxide.
Selhub J, et al., Ann Intern Med. 1999;131(5):331-9.

**TSH**				
TSH		1.10 (mIU/L)	0.50-4.30	NL1
**T3, FREE**				
T3, FREE		4.3 (pg/mL)	3.3-4.8	NL1
**T4, FREE**				
T4, FREE		1.0 (ng/dL)	0.9-1.4	NL1

[redacted] **Page 2 continued on 3**

Accession #: [redacted] **Client Info.** [redacted]
Lab: [redacted]
Patient: [redacted]
Pat. Chart No.: [redacted]
Ordering Phys: [redacted]

GAD65, IA-2, AND INSULIN AUTOANTIBODY

Glutamic Acid Decarboxylase 65 Ab		<5 (IU/mL)	<5	EZ

Note: This test was performed using the GAD65 ELISA method which is standardized against the International reference preparation 97/550.

Ia-2 Antibody		<5.4 (U/mL)	<5.4	EZ

Note: This test was performed using the IA-2 Antibody ELISA method which is standardized against the WHO Reference Reagent 97/550. The reference range reported was established specifically for this test method.

Insulin Autoantibody		<0.4 (U/mL)	<0.4	EZ

C-REACTIVE PROTEIN

C-REACTIVE PROTEIN		0.8 (mg/L)	<8.0	NL1

(CMV) CYTOMEGALOVIRUS ANTIBODIES (IGG,IGM)

Cytomegalovirus Antibody (igg)		<0.60 (U/mL)		NL1

Note:

U/mL	Interpretation
<0.60	Negative
0.60-0.69	Equivocal
> or = 0.70	Positive

A positive result indicates that the patient has antibody to CMV. It does not differentiate between an active or past infection.

Cytomegalovirus Antibody (igm)		<30.00 (AU/mL)		NL1

Note:

AU/mL	Interpretation
<30.00	No Antibody Detected
30.00-34.99	Equivocal
> or = 35.00	Antibody Detected

Results from any one IgM assay should not be used as a sole determinant of a current or recent infection. Because an IgM test can yield false positive results and low level IgM antibody may persist for more than 12 months post infection, reliance on a single test result could be misleading. Acute infection is best diagnosed by demonstrating the conversion of IgG from negative to positive. If an acute infection is suspected, consider obtaining a new specimen and submit for both IgG and IgM testing in two or more weeks.

EPSTEIN BARR VIRUS ANTIBODY PANEL

EBV VIRAL CAPSID AG (VCA)		<36.00 (U/mL)		NL1

Note:

U/mL	Interpretation
<36.00	Negative
36.00-43.99	Equivocal
>43.99	Positive

EBV VIRAL CAPSID AG (VCA)	H	220.00 (U/mL)		NL1

Note:

U/mL	Interpretation
<18.00	Negative
18.00-21.99	Equivocal
>21.99	Positive

EBV NUCLEAR AG (EBNA)	H	>600.00 (U/mL)		NL1

Note:

U/mL	Interpretation
<18.00	Negative
18.00-21.99	Equivocal
>21.99	Positive

[redacted] **Page 3 continued on 4**

Accession #: [redacted] **Client Info.:** [redacted]
Lab: [redacted]
Patient: [redacted]
Pat. Chart No.: [redacted]
Ordering Phys: [redacted]

INTERPRETATION: NL1

Note:
Suggestive of a past Epstein-Barr virus infection.
In infants, a similar pattern may occur as a result
of passive maternal transfer of antibody.

HSV 1/2 IGG,TYPE SPECIFIC AB

HSV 1 IGG, TYPE SPECIFIC	<0.90 (index)		NL1
HSV 2 IGG, TYPE SPECIFIC	<0.90 (index)		NL1

Note:

Index	Interpretation
<0.90	Negative
0.90-1.09	Equivocal
>1.09	Positive

This assay utilizes recombinant type-specific antigens to differentiate HSV-1 from HSV-2 infections. A positive result cannot distinguish between recent and past infection. If recent HSV infection is suspected but the results are negative or equivocal, the assay should be repeated in 4-6 weeks. The performance characteristics of the assay have not been established for pediatric populations, immunocompromised patients, or neonatal screening.

HERPESVIRUS 6 ANTIBODIES (IGG,IGM)

HERPESVIRUS 6 AB (IGG)	H	1:10 (titer)	TXC
HERPESVIRUS 6 AB (IGM)		<1:20 (titer)	TXC
INTERPRETATION		PAST INFECTION	TXC

Note:
REFERENCE RANGE:
IgG <1:10
IgM <1:20

Human Herpesvirus 6 (HHV-6) infects T-lymphocytes, and has been identified as an etiologic agent of exanthema subitum. Rises in antibody titers to HHV-6 have been detected during infection with other viruses. In seroepidemiology studies of the prevalence of exposure using serum screening dilutions of 1:10, the detection of IgG antibody in a mid-life population approaches 100%. Due to this high prevalence of HHV-6 antibody, correlations of single IgG titers with specific diseases are of little clinical value.

Evidence of acute infection or reactivation of HHV-6 is demonstrated by a significant rise or seroconversion of IgG and IgM titers.

This test was developed and its analytical performance characteristics have been determined by Quest Diagnostics Infectious Disease. It has not been cleared or approved by FDA. This assay has been validated pursuant to the CLIA regulations and is used for clinical purposes.

VITAMIN B12

VITAMIN B12	818 (pg/mL)	260-935	NL1

[redacted] **Page 4 continued on 5**

Accession #: [redacted] **Client Info.** [redacted]
Lab: [redacted]
Patient: [redacted]
Pat. Chart No.: [redacted]
Ordering Phys: [redacted]

VITAMIN D,25-OH,TOTAL,IA

VITAMIN D,25-OH,TOTAL,IA	L	25 (ng/mL)	30-100	NL1

```
Note: Vitamin D Status         25-OH Vitamin D:

      Deficiency:                      <20 ng/mL
      Insufficiency:               20 - 29 ng/mL
      Optimal:                   > or = 30 ng/mL

      For 25-OH Vitamin D testing on patients on
      D2-supplementation and patients for whom quantitation
      of D2 and D3 fractions is required, the QuestAssureD(TM)
      25-OH VIT D, (D2,D3), LC/MS/MS is recommended: order
      code 92888 (patients >2yrs).
      See Note 1

      Note 1

      For additional information, please refer to
      http://education.QuestDiagnostics.com/faq/FAQ199
      (This link is being provided for informational/
      educational purposes only.)
```

Performing Laboratory Information:

[redacted]

[redacted] **Page 5 - End of Report**

[redacted] TO: [redacted]

IGeneX Inc. [redacted]

www.igenex.com

Laboratory Director: [redacted]

[redacted]

REFERRING PHYSICIAN

[redacted]

[redacted]

Collected:
Received:
Reported:
Reprinted:
Amended:
Corrected: [redacted]

TEST	SPECIMEN	RESULT	REFERENCE RANGE	UNITS

BORRELIOSIS - Lyme Disease

Lyme Western Blot IgM	Serum			
IGX Criteria:		Negative		
CDC/NYS Criteria:		Negative		

Band (kDa)	18	23-25*	28	30	31*	34*	39*	41*	45	58	66	83-93*
Intensity	-	-	-	-	-	-	-	IND	-	-	-	-

Band Intensity: Positive: + to ++++, Indeterminate: Ind, Negative: (-)

INTERPRETATION	IGX CRITERIA	CDC/NYS CRITERIA
Positive	2 or more of the starred bands are present (+): 23-25*, 31*, 34*, 39*, 41*, 83-93* kDa	2 of the following bands are present (+): 23-25*, 39*, and 41* kDa
Indeterminate	When only bands 31* and 41* kDa or only 31* and 83-93* are present, test 488 is recommended for confirmation	N/A
Negative	If only 41* and 93* kDa are present. Does not meet IGX criteria for a positive or indeterminate test result.	Does not meet CDC/NYS criteria for a positive or indeterminate test result.

Limitation: Bands 31* and 34* kDa are present in Lyme vaccinated patients. Viral antibodies cross react with antigens present at band positions 31* and 83-93* kDa.

[redacted]

Diagnosis should not be based on laboratory results alone. Results should be interpreted in conjunction with clinical symptoms and patient history.

NOTE: Western Blots, ImmunoBlots, Lyme Dot Blot, Epitope, PCR, IFA, FISH, C. pneumoniae IgG/IgA, CD57, IGXSpot, Broad Coverage Antibody, COVID-19 Test - These tests were developed and their performance characteristics determined by IGeneX, Inc. They have not been cleared or approved by the FDA. The FDA has determined that such approval is not necessary. These tests are used for clinical purposes and should not be regarded as investigational or for research. IGeneX, Inc. is licensed by CMS and NYS to perform high complexity clinical laboratory testing.

Page 1 of 4

[redacted]

Collected: [redacted]
Received:
Reported:
Reprinted:
Amended:
Corrected:

TEST	SPECIMEN	RESULT	REFERENCE RANGE	UNITS
Lyme Western Blot IgG	Serum			
IGX Criteria:		**Positive**		
CDC/NYS Criteria:		Negative		

**Band (kDa)**	**18**	**23-25***	**28**	**30**	**31***	**34***	**39***	**41***	**45**	**58**	**66**	**83-93***
**Intensity**	-	-	+	-	-	++	-	+	-	-	-	-

Band Intensity: Positive: + to ++++, Indeterminate: Ind, Negative: (-)

**INTERPRETATION**	**IGX CRITERIA**	**CDC/NYS CRITERIA**
**Positive**	2 or more of the starred bands are present (+): 23-25*, 31*, 34*, 39*, 41*, 83-93* kDa	5 or more of the following bands are present (+): 18, 23-25*, 28, 30, 39*, 41*, 45, 58, 66, 83-93* kDa
**Indeterminate**	Only bands 31* and 41* kDa are present, test 489 is recommended for confirmation	N/A
**Negative**	Does not meet IGX criteria for a positive or indeterminate test result.	Does not meet CDC/NYS criteria for a positive or indeterminate test result.

Limitation: Bands 31* and 34* kDa are present in Lyme vaccinated patients. Viral antibodies cross react with antigens present at band positions 31* and 83-93* kDa.

BABESIOSIS

TEST	SPECIMEN	RESULT	REFERENCE RANGE	UNITS
B. microti IFA - IgM	Serum	<20	< 20 : Negative<br>= 20 : May or may not indicate active infection<br>>=40 : Indicates active infection	Titer

[redacted]

Diagnosis should not be based on laboratory results alone. Results should be interpreted in conjunction with clinical symptoms and patient history.

NOTE: Western Blots, ImmunoBlots, Lyme Dot Blot, Epitope, PCR, IFA, FISH, C. pneumoniae IgG/IgA, CD57, IGXSpot, Broad Coverage Antibody, COVID-19 Test - These tests were developed and their performance characteristics determined by IGeneX, Inc. They have not been cleared or approved by the FDA. The FDA has determined that such approval is not necessary. These tests are used for clinical purposes and should not be regarded as investigational or for research. IGeneX, Inc. is licensed by CMS and NYS to perform high complexity clinical laboratory testing.

Page 2 of 4

[redacted]

[redacted]

Collected: [redacted]
Received:
Reported:
Reprinted:
Amended:
Corrected:

TEST	SPECIMEN	RESULT	REFERENCE RANGE	UNITS
B. microti IFA - IgG	Serum	<40	< 40 : Negative<br>< 160 : May or may not suggest active infection<br>>=160 : Indicates active infection	Titer
Babesia FISH	W blood	Negative		
B. duncani IFA - IgM	Serum	<20	< 20 : Negative<br>= 20 : May or may not indicate active infection<br>>=40 : Indicates active infection	Titer
B. duncani IFA - IgG	Serum	<40	< 40 : Negative<br>< 160 : May or may not suggest active infection<br>>=160 : Indicates active infection	Titer

EHRLICHIOSIS

TEST	SPECIMEN	RESULT	REFERENCE RANGE	UNITS
HME IFA - IgM	Serum	<20	< 20 : Negative<br>= 20 : May or may not indicate active infection<br>>=40 : Indicates active infection	Titer
HME IFA - IgG	Serum	<40	< 40 : Negative<br>< 160 : May or may not suggest active infection<br>>=160 : Indicates active infection	Titer

ANAPLASMOSIS

[redacted]

Diagnosis should not be based on laboratory results alone. Results should be interpreted in conjunction with clinical symptoms and patient history.

NOTE: Western Blots, ImmunoBlots, Lyme Dot Blot, Epitope, PCR, IFA, FISH, C. pneumoniae IgG/IgA, CD57, IGXSpot, Broad Coverage Antibody, COVID-19 Test - These tests were developed and their performance characteristics determined by IGeneX, Inc. They have not been cleared or approved by the FDA. The FDA has determined that such approval is not necessary. These tests are used for clinical purposes and should not be regarded as investigational or for research. IGeneX, Inc. is licensed by CMS and NYS to perform high complexity clinical laboratory testing.

Page 3 of 4

[redacted]

Collected:
Received:
Reported:
Reprinted:
Amended:
Corrected: [redacted]

TEST	SPECIMEN	RESULT	REFERENCE RANGE	UNITS
HGA IFA - IgM	Serum	<20	< 20 : Negative<br>= 20 : May or may not indicate active infection<br>>=40 : Indicates active infection	Titer
HGA IFA - IgG	Serum	<40	< 40 : Negative<br>< 160 : May or may not suggest active infection<br>>=160 : Indicates active infection	Titer
**RICKETTSIOSIS**				
R. rickettsii IFA - IgG	Serum	<40	< 40 : Negative<br>< 160 : May or may not suggest active infection<br>>=160 : Indicates active infection	Titer
R. typhi IFA - IgG	Serum	<40	< 40 : Negative<br>< 160 : May or may not suggest active infection<br>>=160 : Indicates active infection	Titer

End of Report

[redacted]

Diagnosis should not be based on laboratory results alone. Results should be interpreted in conjunction with clinical symptoms and patient history.

NOTE: Western Blots, ImmunoBlots, Lyme Dot Blot, Epitope, PCR, IFA, FISH, C. pneumoniae IgG/IgA, CD57, IGXSpot, Broad Coverage Antibody, COVID-19 Test - These tests were developed and their performance characteristics determined by IGeneX, Inc. They have not been cleared or approved by the FDA. The FDA has determined that such approval is not necessary. These tests are used for clinical purposes and should not be regarded as investigational or for research. IGeneX, Inc. is licensed by CMS and NYS to perform high complexity clinical laboratory testing.

Page 4 of 4

Figure S1. A comprehensive list of the patient's blood test results, including antibody titers.

Supplemental Tables

Table S1. Pro-dopamine regulation pre- and post-behavioral symptoms (Likert Scale 1 - 10). Stopped at week 30 and subsequently began antibacterial therapy.

Days	Depression	Suicide Ideation	Mania	OCD	ADD	Sydenham's Chorea	Anxiety	Agoraphobic	Insomnia	Pain	Body Dysmorphic Disorder	Lethargic	PTSD	Focus
Pre-baseline	10	10	10	10	7	7	10	10	9	8	9	10	10	8
1	7	3	7	6	4	4	7	6	5	8	5	6	6	4
2	7	3	7	5	4	4	7	6	6	8	4	6	6	4
3	7	2	7	6	3	3	7	5	4	8	4	5	5	4
4	7	2	7	6	3	3	7	5	4	8	4	5	5	4
5	7	2	7	6	3	3	7	5	4	8	4	5	5	4
6	7	2	7	6	3	3	7	5	4	8	4	5	5	4
2	7	2	7	6	3	3	7	5	4	8	4	5	5	4
3	7	2	7	6	3	3	7	5	4	8	4	5	5	4
4	7	2	7	6	3	3	7	5	4	8	4	5	5	4
5	7	2	7	6	3	3	7	5	4	8	4	5	5	4
6	7	2	7	6	3	3	7	5	4	8	4	5	5	4
7	7	2	7	6	3	3	7	5	4	8	4	5	5	4
14	7	2	7	6	3	3	7	5	4	8	4	5	5	4
30	7	2	7	6	3	3	7	5	4	8	4	5	5	4
44	7	2	7	6	3	3	7	5	4	8	4	5	5	4
60	7	2	7	6	3	3	7	5	4	8	4	5	5	4
74	7	2	7	6	3	3	7	5	4	8	4	5	5	4
88	7	2	7	6	3	3	7	5	4	8	4	5	5	4
102	7	2	7	6	3	3	7	5	4	8	4	5	5	4

Table S2. Antibacterial pre- and post-behavioral symptoms (Likert Scale 1 - 10). The antibacterial therapy that was tolerated by the patient and prescribed by the attending physician throughout the entire treatment course included Augmentin 875 - 125 mg BID, Bactrim 800 - 160 mg BID, Doxycycline 100 mg BID, and Cefdinir 300 mg BID.

Days	Depression	Suicide Ideation	Mania	OCD	ODD	Sydenham's Chorea	Anxiety	Agoraphobic	Insomnia	Pain	Body Dysmorphic Disorder	Lethargic	PTSD	Focus
Pre-baseline	7	2	7	6	3	3	7	5	4	8	4	5	5	4
1	5	1	4	3	2	2	5	3	2	6	3	3	3	2
2	5	1	4	3	2	2	5	3	2	6	3	3	3	2

Continued

3	5	1	4	3	2	2	5	3	2	6	3	3	3	2
4	4	1	4	3	2	2	4	3	2	6	3	3	3	2
5	4	1	2	2	2	1	4	2	1	3	2	2	2	2
6	4	1	2	2	2	1	4	2	1	3	2	2	2	2
7	4	1	2	2	2	1	4	2	1	3	2	2	2	2
14	4	1	2	2	2	1	4	2	1	3	2	2	2	2
30	4	1	2	2	2	1	4	2	1	3	2	2	2	2
44	4	1	2	2	2	1	4	2	1	3	2	2	2	2
60	4	1	2	2	2	1	4	2	1	3	2	2	2	2
74	3	1	2	2	2	1	3	2	1	2	2	2	2	2
